# The Centesimality of the Fincke High Potencies

**Published:** 1882-05

**Authors:** B. Fincke

**Affiliations:** Brooklyn, N. Y.


### THE CENTESIMALITY OF THE FINCKE HIGH POTENCIES.

B. FINCKE, M. D., BROOKLYN, N. Y.

As the contest about high potencies, generally, is going on in increasing dimensions, it would be a pity to put an end to it, one way or another, since it must, if continuing untrammeled by the powers that be, tend to elucidate the subject more and more, and put it on a secure footing forever more.

If a new idea makes itself felt as being worthy to be introduced into reality, the first resistance it finds is the effort of the opponents to silence it down. If this can no more be done, those opponents themselves keep it before the people by attacking it in every possible manner, fair and foul, and by denouncing and slandering the representants of that idea. If the individuals suffer, they are comforted by the old adage, that "truth will prevail," and why should not the individual be trodden down, if only the truth for which he had been working in his life-time, is raised upon his grave?

Such an idea is the Hahnemannian discovery of potentiation, now about three-quarters of a century old (in 1809 already he speaks of a sixtillionth of a grain), and upheld by a large number of homœopathic physicians who found it to be true in the administration of medicines to the sick. This idea grew out of the fact, that by acting upon a medicine in its crude state in a certain manner, so that to what there is medicinal and curative in it, is given a chance to be distributed throughout a mass of inert vehicle, new medicinal forces are developed according to the degrees of potentiation. This idea then, has been opposed, rejected and neglected by the majority of the homœopathic profession ever since Hahnemann's time when Trinks created the new schism. He (Trinks) and all his followers based their objection upon the "*argumentum ab impossibili,*" and fortified by physical and chemical truth and corresponding mathematical calculations, the schismatics apparently gained ground against the followers of Hahnemann, who base their acceptance of the doctrine of potentiation not upon the dicta of their teacher, but upon facts the most certain, growing out, not of physical and chemical data, and mathematical calculations of various kinds, but out of their own homœopathic science, which teaches them, that the mode of healing, based upon physical and chemical ground, exclusively, is not the true mode of healing, and which furnishes the homœopathic argument. For it is matter of fact, that medicines prepared according to the idea of potentiation, for which enormous quantities of vehicle have been used, and in which the disproportion between the medicine acted on and the vehicle is beyond all human comprehension; it is a fact, I say, that those remedies exert a powerful influence upon both the sick and well human and animal organism. The capable and well-taught homœopathician knows the characteristics of a medicine by its provings upon the healthy, laid down in the Materia Medica Pura, and he therefore knows, when a case comes before him, which remedy he is to apply in order to work a cure. If, now, he applies this remedy in a high potency, and the cure results, it follows, of necessity, that the cause of the cure has been the high potency, for without it, a cure would not have been effected. The observation that this happens in a multitude of cases, and in such cases as the careful physician knows do not get well of themselves, only corroborates the statement and fortifies its correctness.

And *e contrario:* this same high potency applied with all necessary precautions to a healthy human subject of proper sensibility, produces symptoms similar to what it cured in the similarly sick. This is the "*argumentum homœopathicum,*" or "*like cures like.*" Upon this ground, then, the strict followers of Hahnemann have based their mode of treatment, and with perfect right and justice, because they proceed upon scientific principles and rules deducted from actual experiment, pure experience and correct observations withal, called *induction*. About this there is no longer any doubt among the many who daily use high potencies in their practice.

Leaving aside, then, what on the part of the natural sciences has been claimed as justifiable opposition to high potencies, as not properly belonging to medical science, *per se*, the efforts to draw the subject in question out of its legitimate sphere into that of general natural science, which just now is domineered over by the materialistic school, must be respectfully declined. If the natural sciences have not so far advanced as to recognize the reality of homœopathic high potencies, it is no fault of the science of homœopathics. If *they* cannot assign the quantities developed from well-known substances, because they are infinitesimal, and defy the finest chemical and physical tests, though being capable of mathematical demonstration, it is no fault of ours. For *we* homœopathicians can assign them, we know that this is such and such a potency which we apply and we know beforehand what it will do, and we assign their distinct action by means of our art of healing. And there comes a powerful ally in one of the most eminent German scholars, Prof. Dr. G. Jæger, who shows the action of high potencies by his *Neural Analysis* already as high as 4,000 centesimal, and by my electro-magnetic method, the action of any high potency upon a sensitive person can be seen in a few minutes.

Therefore, the attempts to ridicule and despise the high potencies for their want of sufficient magnitude, appears to be idle when contrasted with the practical working of this matter in our homœopathic profession, not to speak of the nature of motion which, though of metaphysical origin, governs, nevertheless, all the palpable chemical and physical processes, large and small, and that according to homœomatic laws. And ridicule comes home to roost sooner or later.

All that, for practical purposes, is desired with regard to high potencies, is the confidence we must have in them that they are so prepared, that the medicine needed will be pure and uncontaminated, from the lowest to the highest potencies, and that it is the potency indicated on the label. In this respect the scale plays an important part as is self-evident.

I, for my part, have adopted the Hahnemannian scale for my high potencies which is the centesimal. And so I have declared in all my writings and sayings in and out of the American Institute of Homœopathy. Now, of late, several mathematicians, of more or less note, have doubted the correctness of my statement to which, surely, they have a perfect right. But I am sorry to say, that their views do not stand the test of rigorous examination. I do not say that their calculations are incorrect, but I do say, that their premises are so, and that they worked upon misconceived data. Moreover, they present the incomparable feat of making a second step before the first has been made, and time does not trouble them at all. I, for one, nevertheless, hailed the time when such calculations began to appear as a sign of progress of the cause of high potencies, and with it of homœopathics, because, at any rate, it shows the interest taken in these matters. But as discredit has been thrown upon the centesimality of my preparations, it has placed me in a false position which, I hope, the profession will allow me to rectify.

Homœopathic physicians in active practice cannot be expected to be highly educated mathematicians. I hope, therefore, to be excused, if I do not enter the arena where these gifted few break their lances. But the following few remarks will be sufficient, I think, to prove to any one, that my high potencies are really centesimal, and therefore in accordance with Hahnemann's scale, though prepared by a different process.

If you take a vial capable of holding one fluidrachm, including the end of the syphon, and place into it the one hundredth part of one fluidrachm of medicine and add in proper time and manner, by syphon, ninety-nine hundredths of a fluidrachm of the vehicle, there can be no doubt but that, provided the one hundredth fluidrachm is so prepared as to be in proper time distributable throughout the ninety-nine hundredths of a fluidrachm of vehicle, this one hundredth fluidrachm is distributed throughout that quantity of vehicle and that every part of the one hundredth fluidrachm of the mass now contains the one hundredth part of the original one hundredth fluidrachm applied. This, therefore, is the first potency, and it is just as centesimal as Hahnemann's own, obtained by dropping one drop of the medicine into ninety-nine drops of alcohol, and shaken twice or oftener.

In order to prepare the second potency, the fluxion proceeds until the vial is filled a second time with a fluidrachm of vehicle, and after that only a repetition of the occurrence with the first filling of the vial will have taken place. Now, if in the first instance in the fluidrachm of the first potency were contained the one hundredth part of one hundredth fluidrachm of medicine in every one hundredth fluidrachm of vehicle, we have now one hundredth part of one hundredth fluidrachm or $\frac{\frac{1}{100}}{100}$ of the first potency in every hundredth drachm of vehicle which, of course, amounts to one ten thousandth part in every one hundredth fluidrachm of vehicle, or to the second potency, and this is just as surely the second centesimal potency, as Hahnemann's own, obtained by dropping one drop of the first potency into ninety-nine drops of alcohol, and shaking it twice or more. And so indefinitely.

The following is an expression of the fluxion process in numbers, conceived in 100 parts of .01 fl ʒ each, for every fluidrachm.

Let the starting-point of potentiation be .01 fl. ʒ of the sixth centesimal potency, made by hand, called the mother potency, resting upon the bottom of the potentiating vial, holding, with the syphon, one fluidrachm. The vehicle enters by a fine opening controlling the thoroughness of the process at the bottom of the vial in the ratio of 500 fl. ʒ per hour, and mingles with the .01 fl. ʒ of the sixth potency in the following manner:

1) $.01^{6}$ fl. ʒ pot. + .01 fl. ʒ veh. = $.02^{6.01}$ fl. ʒ pot.; ratio 1 : 2.

2) $.02^{6.01}$ fl. ʒ " + .01 " " = $.03^{6.02}$ fl. ʒ " " 1 : 3.

3) $.03^{6.02}$ fl. ʒ " + .01 " " = $.04^{6.03}$ fl. ʒ " " 1 : 4.

and so on till .99 fl. ʒ of vehicle are added in continuous flux to the original .01 fl. ʒ of the sixth potency; we then have the sixth and ninety-nine hundredth potency. The last .01 fl. ʒ vehicle flowing in makes the seventh potency full, for we have

100.) $.99^{6.99}$ fl. ʒ pot. +.01 fl. ʒ veh. = $1^{7}$ fl. ʒ pot.; ratio 1 : 100.

The next .01 fl. ʒ vehicle entering below leaves no room for the topmost $.01^{7}$ fl. ʒ potency in the vial, consequently, according to Bacmeister's remark, it must flow over whilst the .01 fl. ʒ vehicle succeeding the .01 fl. ʒ which finished the seventh potency below continues the potentiation. At the moment when this happens, when the last .01 fl. ʒ vehicle has entered below, and the first $.01^{7}$ fl. ʒ potency is going over, the whole vial contains one fluidrachm of the seventh centesimal potency, and the ratio is 1 : 100 as in Hahnemann's process.

If, now, the fluxion continues, the next .01 fl. ʒ of vehicle entering the vial, mingles with the $.01^{7}$ fl. ʒ potency contiguous to it, and the .02 fl. ʒ of the mixture are converted into the $.02^{7.01}$ fl. ʒ potency, for we have

1.) $.01^{7}$ fl. ʒ pot. + .01 fl. ʒ veh. = $.02^{7.01}$ fl. ʒ pot., ratio 1 : 2.

2.) $.02^{7.01}$ fl. ʒ " + .01 " " = $.03^{7.02}$ " " " 1 : 3.

and so on, till again .99 fl. ʒ vehicle have flown in, which makes the seven and ninety-ninth potency, and with the next .01 fl ʒ vehicle entering, completes the eighth potency. For we have

100.) $.99^{7.99}$ fl. ʒ pot. + .01 fl. ʒ veh. = $1^{8}$ fl. ʒ pot., ratio 1 : 100.

Then the next .01 fl. ʒ vehicle makes the topmost .01 fl. ʒ of the eighth potency flow out, and continues the potentiation.

In this manner the potentiation by fluxion goes on indefinitely, and if started aright in the ratio of 1 : 100, it necessarily must continue so to the end.

It is evident, then, that the amount of vehicle flown through the vial must be measured by the number of fluidrachms used. It is also evident, that the vial serving as the receptacle for potentiation, must hold just one fluidrachm, if .01 fl. ʒ of potency has been used to begin with.

The notation according to powers is as justified as that in the Hahnemannian process, and remedies obtained by either process may well be compared, if only the necessary centesimal scale is preserved. But this comparison, practically, stops short at the 60th potency, because Hahnemann, as far as known, has prepared none higher, and no other potencies higher than this are known to have ever been made by dropping the one drop of medicine into ninety-nine drops of alcohol. Those who call the fluxion process bottle-washing, betray their gross ignorance. If they clean their bottles by the fluxion process they will be dirty enough, and not fit for a fluxion process for potentiation.

Therefore, the objections that Fincke's high potencies are not centesimal, but "unesimal," or that there is nothing in them after a while, and a short while at that, and that they do not compare with Hahnemann's potencies, or that they are lower than these, and as their labels indicate, fall simply to the ground.

In this connection I must protest against the arbitrary procedure of certain editors and writers, who without ceremony reduce the number of the potencies in their publication of clinical cases and provings and omit the mark of the potentiator altogether. It is clear that there must be a difference in the various preparations of Hahnemann, Korsakoff, Jenichen, Rentsch, Lentz, Petters, Lappe, Lehrmann, Dunham, Schwabe, Zahn, Seeger, Zennegg, Hess, Tafel,

Boericke, Swan, Skinner, Deschere and others I am not aware of besides myself, and scientific accuracy and common justice and courtesy demand that the high potencies they have made should be marked as they mark them. Or else the object of the publication to serve as testimony for the efficacy of Homœopathy and her high potencies is lost. It also creates the false impression, that the potencies were of the writer's own manufacture.

The question, however, how it is that the medicine be distributable in a quantity of inert vehicle to an incredible extent cannot now here be discussed, and requires long-continued investigations to which the researches in natural science of later times form no inconsiderable contributions. But in as much as they do not acknowledge any reality of our high potencies beyond the 11th centesimal potency, very little can be expected from that quarter for the solution of the problem of potentiation.

*November*, 14*th*, 1880.

